# Light input pathways to the circadian clock of insects with an emphasis on the fruit fly *Drosophila melanogaster*

**DOI:** 10.1007/s00359-019-01379-5

**Published:** 2019-11-05

**Authors:** Charlotte Helfrich-Förster

**Affiliations:** Neurobiology and Genetics Biocentre, University of Würzburg, 97074 Würzburg, Germany

**Keywords:** Photoreception, Photopigment, Cryptochrome, Rhodopsin, Compound eyes, Entrainment

## Abstract

Light is the most important Zeitgeber for entraining animal activity rhythms to the 24-h day. In all animals, the eyes are the main visual organs that are not only responsible for motion and colour (image) vision, but also transfer light information to the circadian clock in the brain. The way in which light entrains the circadian clock appears, however, variable in different species. As do vertebrates, insects possess extraretinal photoreceptors in addition to their eyes (and ocelli) that are sometimes located close to (underneath) the eyes, but sometimes even in the central brain. These extraretinal photoreceptors contribute to entrainment of their circadian clocks to different degrees. The fruit fly *Drosophila melanogaster* is special, because it expresses the blue light-sensitive cryptochrome (CRY) directly in its circadian clock neurons, and CRY is usually regarded as the fly’s main circadian photoreceptor. Nevertheless, recent studies show that the retinal and extraretinal eyes transfer light information to almost every clock neuron and that the eyes are similarly important for entraining the fly’s activity rhythm as in other insects, or more generally spoken in other animals. Here, I compare the light input pathways between selected insect species with a focus on *Drosophila’s* special case.

## Introduction

Virtual all organisms possess endogenous circadian clocks. These enable them to be prepared in advance for the cyclic 24-h changes in the environment, instead of merely responding passively to them. Needless to say that the circadian clocks themselves have to be synchronized (= entrained) to the external 24-h cycles in order to work as proper daily clocks. Most organisms use changes in the quantity and quality of light around dawn and dusk as their primary *Zeitgeber* for ‘photoentrainment’ (Roenneberg and Foster 1997). The detection of changes in irradiance and spectral light composition is qualitatively different from the fine spatial and temporal resolution carried out by the photoreceptors in the eyes that are involved in image formation. Therefore, most if not all animals possess special ‘circadian’ photopigments in or outside their eyes that fulfil this task (reviewed by Doyle and Menaker 2007). Cryptochrome (CRY) is such a photopigment that is expressed in every clock cell (even in the photoreceptor cells of the eyes) and can entrain the circadian clocks in the brain and peripheral organs of translucent animals such as fruit flies (Emery et al. 2000). CRY or other cellular photopigments can also entrain peripheral clocks of translucent zebra fish (Whitmore et al. 2000). Other circadian cellular photopigments are the so-called deep brain photoreceptors (different nonvisual opsins) of non-mammalian vertebrates (Davies et al. 2015; Hang et al. 2016). In mammals, functional deep brain opsins have so far not been identified, but melanopsin in a subset of the retinal ganglion cells fulfils the role as circadian cellular photopigment (Provencio et al. 1998; Berson et al. 2002; Hattar et al. 2002; reviewed in Lazzerini Ospri et al. 2017). All these photopigments appear to convey information about environmental light conditions to the circadian clock and to mediate photoentrainment and/or photoperiodic responses.

Most importantly, however, the circadian cellular photopigments do not work in isolation. The eyes contribute to circadian entrainment. For example, mammals are only ‘circadianly blind’ (do not entrain to external light–dark cycles) when melanopsin and the rhodopsins of rods and cones are gone (Hattar et al. 2003; Güler et al. 2008). This is because the rods and cones signal to the melanopsin-positive ganglion cells and the latter then signal via the retino-hypothalamic tract to the circadian master clock in the suprachiasmatic nuclei (SCN) of the hypothalamus (McNeill et al. 2008). Thus, melanopsin-positive ganglion cells integrate the light signals coming from the rods and cones with the ones coming from melanopsin. Similarly, fruit flies are only ‘circadianly blind’ when CRY and all six rhodopsins are gone (Helfrich-Förster et al. 2001). Here, the photoreceptor cells of the eyes signal to the circadian pacemaker neurons (Li et al. 2018) of which many contain CRY (Yoshii et al. 2008; Benito et al. 2008). Thus, the light signals coming from the eyes are integrated with the ones coming from CRY within the circadian pacemaker neurons themselves. There is even evidence for a retrograde signalling from the melanopsin-positive ganglion cells of mice and CRY of flies to the photoreceptor cells in the eyes affecting light sensitivity and/or adaptation of the latter (Mazzotta et al. 2013; Prigge et al. 2016; Schlichting et al. 2018). Furthermore, studies in mice indicate that melanopsin contributes to the representation of images in the early visual system (Allen et al. 2017). Hence, cellular photopigments and eyes interact in manifold ways. The degree of interaction between eyes and cellular photopigments most certainly depends on the specific niche occupied by the animal and is, therefore, expected to be different in diverse species.

Insects are especially interesting in this respect, because they represent an abundant diverse species group that is distributed all over the world and adapted to very different habitats. In addition, their photobiology is interesting, since besides cellular extraretinal photopigments, most adult insects possess several eyes: two large compound eyes, ~ 3 ocelli at the top of their head and sometimes remnants of their larval stemmata that are maintained and even restructured during development (e.g. Fleissner et al. 1993; Helfrich-Förster et al. 2002; Sprecher and Desplan 2008).

Here, I will address the following key questions in selected insect species. (1) What is the functional connection between the eyes and the circadian clock in the brain? (2) What is the relative contribution of the eyes to photoentrainment of the clock? (3) Which cellular circadian photopigments are present and how do they interact with the eyes? I will start with insects that possess a mammalian-like molecular clockwork, such as cockroaches, crickets, beetles and bees. These insects appear to possess light-insensitive forms of CRY that are part of the core clock and do not work as photopigments (see Yuan et al. 2007; Sandrelli et al. 2008; Tomioka and Matsumoto 2010 for reviews). For these insects, the compound eyes are very important for entraining the clock and there is so far no evidence for deep brain photoreceptors, probably because most of them have strongly pigmented head capsules that prevent light from coming through. Nevertheless, several of these insects have extraretinal photoreceptors close to their eyes or underneath translucent windows in their cuticle. The second group of insects possesses both forms of CRY, light-sensitive and light-insensitive ones. Usually these insects have less pigmented head capsules that might be transparent for light and appear to possess deep brain photoreceptors (Zhu et al. 2005; Cortés et al. 2010). To these insects belong aphids, moths, butterflies and mosquitoes. I will end my review with discussing flies that possess only the light-sensitive form of CRY and appear to have directly light-entrainable circadian clocks throughout their body and head (e.g. Plautz et al. 1997; Ivanchenko et al. 2001).

## Photoentrainment in cockroaches, crickets, beetles, bees and ants

### Cockroaches

**Fig. 1:**
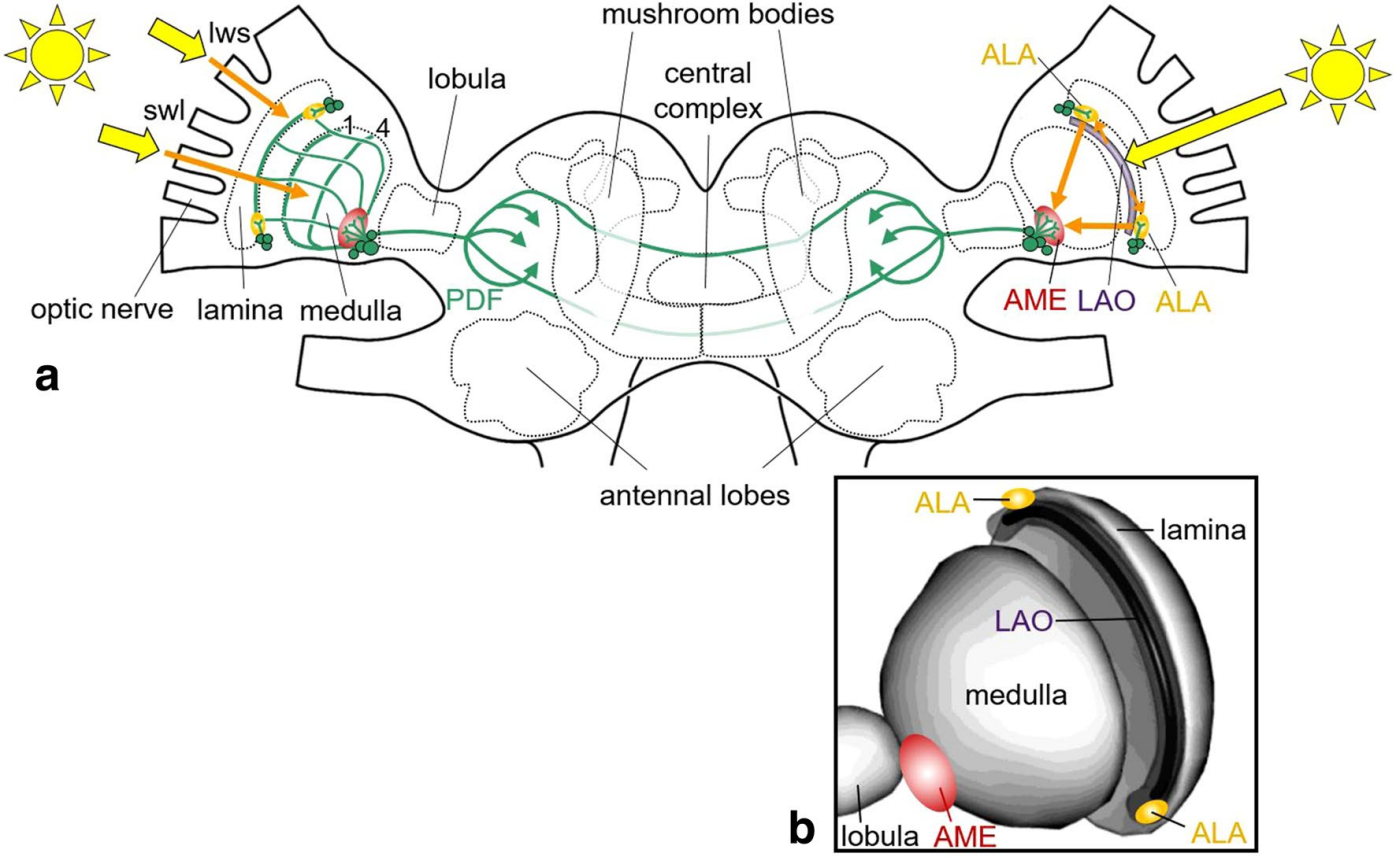
Rough schematic representation of the light input pathways in the cockroach. **a** Scheme of the *Rhyparobia madeira* brain with the principal arborizations from the pigment-dispersing factor (PDF)-positive clock neurons (green). Only a few PDF neurons are exemplary shown. Most of them are close to the accessory medulla (AME) and invade it; fewer are close to the accessory laminae (ALA) and invade these. Note that PDF-positive fibres connect the AME and ALA. Light reaches the circadian clock neurons in the AME through the compound eyes (left) and putatively via the lamina organs (LAO) (right). Photoreceptor cells in the LAO project to the two ALAs (orange small arrows) and from the ALAs to the AME. The PDF fibres are omitted for clarity in the right medulla and lamina and only shown in the left optic lobe. There, they invade the proximal layer of the lamina and layers 1 and 4 of the medulla. Light from the compound eyes may reach the PDF-positive fibres in the lamina via the long-wavelength sensitive short photoreceptor cells (lws) that are mainly responsive to green light. In addition, light may reach the PDF neurons indirectly via the short-wavelength sensitive long photoreceptor cells (swl) that respond to UV and terminate in medulla layer 2. This figure is redrawn from Wei et al. (2010) and Stengl and Arendt (2016) with information added from Giese et al. (2018) and Fleissner et al. (2001). **b** Three dimensional representation of the lamina organ (LAO). Modified from Fleissner et al. (2001). Labelling as in **a**

The cockroaches *Leucophaea maderae* and *Periplaneta americana* were the first insects, in which a circadian master clock was successfully localized to a specific area in the optic lobe with the help of lesions (Nishiitsutsuji-Uwo and Pittendrigh 1968; Roberts 1974; Sokolove 1975). This area is situated close to the second optic ganglion, the medulla, and many years later, it was characterized in detail by immunohistochemical studies in *Leucophaea maderae* (now renamed into *Rhyparobia maderae*) (Homberg et al. 1991; Stengl and Homberg 1994; Petri et al. 1995; Reischig and Stengl 2003). The cockroach circadian pacemaker centre turned out to be located in a small neuropil of ovoid shape—the accessory medulla (AME) (Fig. 1). The AME is densely innervated by ~ 240 peptidergic and GABAergic neurons and is organized into a core that receives photic input and a shell, from which output neurons enter the central brain (reviewed in Stengl et al. 2015; Stengl and Arendt 2016). Thus, from its organization, the AME largely resembles the mammalian SCN. The best-characterized neurons in the AME of the Madeira cockroach express the neuropeptide pigment-dispersing factor (PDF) (Reischig and Stengl 1996, 2003). These PDF-positive neurons have an especially prominent role in the circadian system. They comprise local neurons, output neurons and even light input neurons (Stengl and Arendt 2016). Besides its roles as circadian input and output factor, PDF is crucial for the synchronization of molecular and membrane potential oscillators within and between circadian clock neurons (Schneider and Stengl 2005; Stengl et al. 2015). Most interestingly, PDF has a mammalian functional analogue in the vasoactive intestinal polypeptide (VIP) (Hastings et al. 2014; Pauls et al. 2014). PDF is not only expressed in the AME of cockroaches but also in their accessory laminae (ALA) that lie dorsally and ventrally of the lamina as was shown for the Madeira cockroach, the German cockroach (*Blattella germanica*) and the double-striped cockroach (*Blattella bisignata*) (Wen and Lee 2008; Stengl and Arendt 2016) (Fig. 1). While PDF neurons in the AME are involved in the control of behavioural rhythmicity, the PDF neurons of the accessory laminae project to the AME and appear to carry light information to the circadian master clock (Lee et al. 2009; Stengl and Arendt 2016; Giese et al. 2018; see below).

**Fig. 2:**
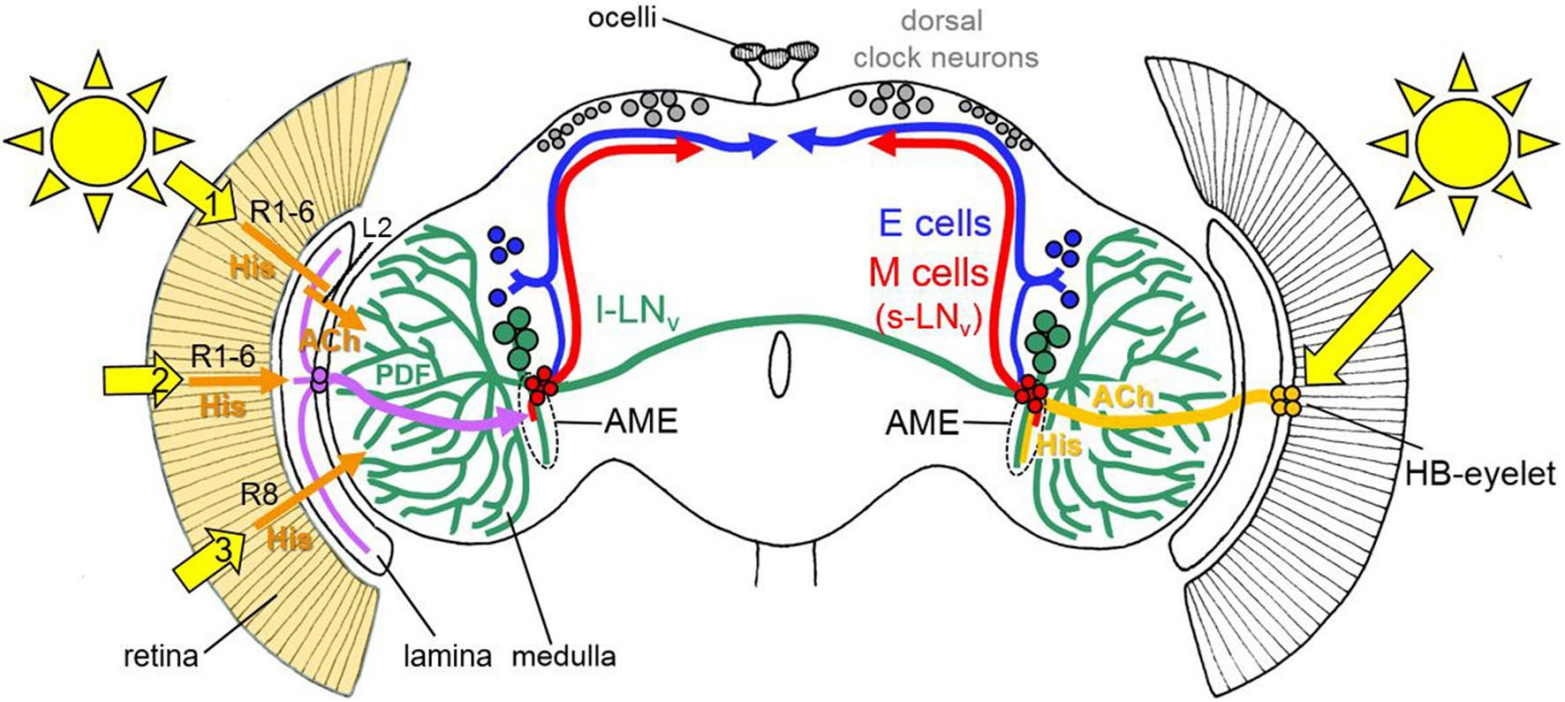
Rough schematic representation of the light input pathways from the eyes in *Drosophila melanogaster*. Light reaches the circadian lateral clock neurons [M cells (= s-LN_v_), E cells (mainly LN_d_), and the large ventrolateral neurons (l-LN_v_)] through the compound eyes (left) and the Hofbauer–Buchner (HB)-eyelets (right). All receptor cells of the compound eyes use histamine (His) as a neurotransmitter, whereas the HB eyelets utilize histamine and acetylcholine (ACh). The HB eyelets project into the accessory medulla (AME) and signal via histamine to the l-LN_v_ and via ACh to the M cells. The l-LN_v_ and the M cells (s-LN_v_) express the neuropeptide PDF (pigment-dispersing factor). The PDF fibres are indicated in green and red. From the compound eyes, there are three putative input pathways to the clock neurons. In the first one (1), receptor cells 1–6 (R1–6) signal via His to the lamina monopolar cells (L2). L2 cells express ACh and signal in the distal medulla to the l-LN_v_. In the second one (2), R1-6 signal to wide-field fibres arborizing in the lamina and stemming from two peptidergic interneurons (AstC/CcapR in lilac) that are located between lamina and medulla (Li et al. 2018). These neurons send axons into the AME, where they contact most clock neurons. In the third light-input pathway (3), Rh6-positive R8 cells that appear to play an integrative role in the light input from all other receptor cells, signal indirectly to the circadian clock neurons (Alejevski et al. 2019). The exact connections are, however, still unknown. Putative light input signals from the ocelli are omitted. Modified from Senthilan et al. (2019)

Cockroaches possess three ocelli besides their compound eyes, but only lesions of the compound eyes abolished the entrainment of locomotor activity rhythms to light–dark cycles, which indicates that the compound eyes are the only photoreceptors that synchronize the circadian clock (Roberts 1965; Nishiitsutsuji-Uwo and Pittendrigh 1968; Page et al. 1977; Page and Barrett 1989). The eyes are indirectly connected to the AME via not yet completely identified pathways, in which several neurotransmitters and neuropeptides, including PDF, are involved (Schendzielorz and Stengl 2014; Arendt et al. 2017; Giese et al. 2018). As many insects, the cockroach compound eye possesses long- and short-wavelength sensitive photoreceptor cells that use histamine as neurotransmitter (Loesel and Homberg 1999). The first type is mainly sensitive to green light and sends short axons into the lamina (Fig. 1). This type might contact PDF fibres that arborize in the proximal layer of the lamina and may stem from the PDF neurons of the AME or the ALA. The second type is mainly sensitive to UV light and sends axons in medulla layer 2, which is devoid of PDF fibres (Fig. 2). Nevertheless, there are several interneurons that connect the two medulla layers and may confer light information to the PDF neurons of the AME.

The above-mentioned ALA appear to play a prominent role in the light input pathway to the clock. The ALA are assumed to be innervated by extraretinal photoreceptor axons of the lamina organ, an elongated structure adjacent to the anterior edge of the lamina that expresses CRY (Petri et al. 1995; Fleissner et al. 2001) (Fig. 1). The lamina organ may serve as extraocular photoreceptor for light entrainment of the circadian clock as was suggested before for beetles (Fleissner et al. 1993, see below). Thus, even cockroaches may possess cellular photopigments in an organ very close to the eyes that may contribute to circadian photoreception. The close vicinity to the eyes might have obscured their existence in the older lesion studies, because the latter might have destructed the extraretinal photoreceptors as well. Nevertheless, why the lamina organ expresses CRY and how this presumably light-insensitive mammalian-type CRY can contribute to circadian photoreception remains to be clarified. It is also possible that cockroaches possess light-sensitive CRY in addition to light-insensitive CRY. So far, the cockroach genome is not completely sequenced and the light sensitivity of cockroach CRY was not directly tested. Thus, we cannot completely exclude this possibility. This is also true for crickets, beetles, bees and ants that are treated in the following chapters.

### Crickets

The crickets, *Gryllus bimaculatus*, *Teleogryllus commodus*, *Achaeta domesticus*, *Hemideina thoracica*, *Modicogryllus siamensis*, *Gryllodes sigillatus* and *Dianemobius nigrofasciatus*, have been extensively studied for their locomotor activity and/or singing (stridulatory) rhythms (reviewed by Tomioka 2014). Similar to cockroaches, the circadian master clock of *G. bimaculatus, T. commodus, H. thoracica*, *G. sigillatus* and *D. nigrofasciatus* could be localized to the optic lobes, and the compound eyes turned out to be the major circadian photoreceptors in most of these species (Sokolove and Loher 1975; Tomioka and Chiba 1984, 1986; Tomioka et al. 1990; Waddell et al. 1990; Yukizane and Tomioka 1995; Abe et al. 1997; Shiga et al. 1999). Nevertheless, the role of the AME and the PDF neurons in the circadian system is less clear in crickets as compared to cockroaches. In *G. bimaculatus*, the partial destruction of the optic lobes led to arrhythmic locomotor activity even when the AME and the PDF neurons remained intact, suggesting that the PDF neurons in the AME alone are not sufficient for controlling activity rhythms (Okamoto et al. 2001). A knockdown of PDF by RNA interference shortened this crickets’ free-running period, reduced their nocturnal activity and affected their photic entrainment, but did not abolish circadian rhythmicity (Saifullah and Tomioka 2003; Abdelsalam et al. 2008; Hassaneen et al. 2011). This indicates that PDF is involved in photic entrainment and fine-tuning of the free-running period of the circadian clock, perhaps by coupling different clock neurons, but that it appears not essential for rhythmic behaviour. It is also not clear whether the AME plays a similar important role as master clock in *G. bimaculatus* as it does in cockroaches, although the medulla is the target of neurons that connect the bilateral optic lobe master clocks (Yukizane et al. 2002) and cockroaches possess a pronounced AME (Homberg et al. 1991). Crickets may possess several clock centres that control rhythmicity and determine period via parallel clock output pathways. Only one of these may locate in the AME and use PDF as output, others close to the ALA and/or in the central brain and work without PDF (Helfrich-Förster 2005; Tomioka 2014). Although the location and organization of the master clock are less clear in crickets, the pathway for photic entrainment in crickets shows large similarities to cockroaches.

As in cockroaches lesions of the compound eyes but not the ocelli impaired photoentrainment of the activity rhythms of *G. bimaculatus* (Tomioka and Chiba 1984; Yukizane and Tomioka 1995), suggesting that the compound eyes contain the sole photoreceptors for entraining the circadian clock. Recent studies showed that circadian entrainment is mediated by green-sensitive opsins in the compound eyes (Komada et al. 2015). Their activation leads to an increase of the bZip transcription factor c-fos in the optic lobes that resets the circadian clock via CRY (Kutaragi et al. 2018). The down-regulation of the *cry* and *c*-*fos* genes by RNAi strongly disturbs photoentrainment showing that these factors are critically involved in circadian photoreception (Kutaragi et al. 2018). Most interestingly, CRY and c-fos are expressed in an area close to the lamina (Kutaragi et al. 2018) that resembles the cockroach lamina organ and that was supposed to act as extraretinal photoreceptor organ (see above). In *G. bimaculatus*, there is so far no evidence that this putative lamina organ can entrain locomotor activity rhythms in absence of the compound eyes, but some indication for extraretinal photoreception comes from the band-legged ground cricket, *Dianemobius nigrofasciatus* (Shiga et al. 1999). After removal of both compound eyes and all ocelli in this cricket, some animals still entrained to light–dark cycles. Histological examination of the operated crickets revealed that parts of the lamina remained intact after the surgery. Furthermore, Figure 2 B in Shiga et al. (1999) shows a small structure anterior to the lamina resembling the putative lamina organ of *G. bimaculatus* that survived the surgery. Thus, entrainment might have occurred via extraretinal photoreception. Similarly, crickets of the species *Hemideina thoracica* remained entrained after surgical removal of the retinae of both compound eyes, once more suggesting that extraretinal photoreception, perhaps via lamina organs, contributes to photic entrainment in crickets (Waddell et al. 1990).

### Beetles

The so far best anatomical description of the lamina organs exists for the carabid beetle, *Pachymorpha sexguttata* and the tenebrionid beetle, *Zophobas morio* (Fleissner et al. 1993). In both beetles, these organs have an elongated structure, and are about 20–40 µm wide and more than 300 µm long. They are situated at the fronto-dorsal rim of the laminae beneath window-like thinnings of the cuticle. They are highly organized and composed of sheath cells that lack shielding pigments and receptor cells that contain rhabdom-like structures. The rhabdom-like structures express rhodopsin and retinal S-antigen (arrestin), which is typical for photoreceptor cells. Axons arising from the receptor cells run into adjacent accessory laminae, which connect to the AME as already described for cockroaches. The master clock of beetles has previously been localized to the optic lobe in *Blaps gigas* (Koehler and Fleissner 1978), *Pachymorpha sexguttata* (Fleissner 1982) and *Carabus problematicus* (Balkenohl and Weber 1981). Immunocytochemical studies in *P. sexguttata* showed that neurons in the AME express the clock protein Period (PER) and the neuropeptide PDF (Frisch et al. 1996) making it very likely that the AME contains the master clock as demonstrated above for cockroaches. The two bilateral master clocks of beetles are only weakly coupled with each other, because bilateral neuronal connections appear virtually absent. Consequently, the circadian rhythms generated in each AME can easily be desynchronized from each other by differently illuminating the two compound eyes as shown in *B. gigas* (Koehler and Fleissner 1978). This strongly indicates that photoentrainment occurs via the ipsilateral compound eyes or the adjacent extraretinal lamina organs. Beetles have usually no ocelli, but they possess metamorphosed larval stemmata attached to the posterior sides of the optic lobes that might be still light sensitive (Fleissner et al. 1993). Thus, circadian photoreception can theoretically occur via the compound eyes, the lamina organs and the stemmata, although photoreception via the stemmata appears unlikely, because these lie underneath a thick presumably light-tight cuticle.

### Bees and ants

Like in cockroaches, crickets and beetles, only the light-insensitive form of CRY was found in bees and ants. Therefore, they are supposed to entrain their circadian rhythms mainly through the compound eyes. So far, no lamina organ has been detected in their brain that could contribute to photoentrainment. However, honey bees appear to possess a vertebrate-like deep brain opsin, called pteropsin (Velarde et al. 2005). Pteropsin is expressed in 12 neurons that are located in the same place in the lateral brain in which Period and PDF-expressing neurons have been identified (Fuchikawa et al. 2017; Beer et al. 2018), suggesting that the circadian clock neurons of the bee are light sensitive per se. Whether this is true has to be shown in future studies. For ants, nothing is known about extraretinal photoreception, but the organization of the circadian system of *Camponotus floridanus* appears very similar to that of honey bees (Kay et al. 2018).

## Photoentrainment in moth, butterflies, aphids and mosquitoes

These diverse groups of insects possess for sure two forms of CRY, the light-insensitive and light-sensitive one. Furthermore, all these insects own deep brain photoreceptors, either for entraining their circadian clock or for measuring day length to time photoperiodic annual responses (e.g. diapause). Photoperiodic responses appear also to depend on circadian photoreception, but they are independent from circadian entrainment. For example, circadian entrainment is difficult to assess in species that move very little, such as aphids (see Beer et al. 2017; Joschinski et al. 2016); nonetheless, aphids show strong photoperiodic responses in response to changes in day length.

In a legendary series of experiments, Truman and Riddiford demonstrated that the circadian master clock controlling flight activity and eclosion of the silk moths, *Antheraea pernyi* and *Hyalophora cecropia*, resides in the central brain and is entrained to light–dark cycles by deep brain photoreceptors (Truman and Riddiford 1970; Truman 1972, 1974). Many years later, Reppert and coworkers characterized the molecular components of the *A. pernyi* clock and found that this silk moth possesses mammalian-type and *Drosophila*-type clock genes and proteins (Chang et al. 2003). For example, *A. pernyi* owns the *Drosophila*-like Timeless protein (TIM) that can interact with light-activated CRY leading to its degradation. Thus, it is likely that the silk moth clock neurons are intrinsically light sensitive as it is the case in *Drosophila*. In contrast to the so far discussed insects, the silk moth master clock appears to be located in the dorsal brain and not in the AME without any direct neuronal connection to the compound eyes (Sauman and Reppert 1996; Sehadová et al. 2004). Similarly, the master clock of the monarch butterfly, *Danaus plexippus,* lies in the dorsal brain, co-expresses *Drosophila*-like CRY and TIM and is light-sensitive (Zhu et al. 2008). A brain-centred photoreceptor has also been implicated in the photoperiodically controlled termination of diapause in *A. pernyi* (Williams and Adkisson 1964) and in diapause induction of *Pieris brassicae* (Seuge and Veith 1976).

In the aphid *Megoura viciae*, the site of photoreception for initiating sexual morphs under decreasing photoperiods in late summer has also been localized to the dorsal brain (Lees 1964). More recent studies in the pea aphid, *Acyrthosiphon pisum*, identified the clock genes and revealed that *A. pisum* possesses also mammalian-type and *Drosophila*-type clock genes that are expressed in the dorsal brain, but additionally also in the lateral brain (Cortés et al. 2010; Barberà et al. 2017). The location of the master clock in mosquitoes is less well studied. There is just one report of Kasai and Chiba (1987) showing that *Culex pipiens* still show light-entrainable flight rhythmicity after ablation of their optic lobes, indicating that circadian photoreceptors are located in the central brain. As moth, butterflies and aphids, mosquitoes possess mammalian-type and *Drosophila*-type clock genes (Gentile et al. 2009; Meuti et al. 2015).

## Photoentrainment in flies

The best-characterized circadian clock is that of the fruit fly *Drosophila melanogaster* (reviewed by Helfrich-Förster 2017; Top and Young 2018), but other higher fly species appear to have a comparable clock organization with some differences existing within the Drosophiliids (Codd et al. 2007; Muguruma et al. 2010; Menegazzi et al. 2017; Beauchamp et al. 2018; Bertolini et al. 2018; Helfrich-Förster et al. 2018). Furthermore, all higher flies appear to possess only the light-sensitive form of CRY that may contribute to entrainment (An et al. 2004; Fuchikawa et al. 2010; Bertolini et al. 2018) and is generally regarded as the main circadian photopigment of *D. melanogaster* (Stanewsky et al. 1998; Emery et al. 2000). In addition, some fly species possess extraretinal eyelets that are metamorphized larval eyes, and like the stemmata of beetles, they are located in a posterior position of the optic lobes (Hofbauer and Buchner 1989; Malpel et al. 2002; Helfrich-Förster et al. 2002; Sprecher and Desplan 2008). In the following, I will describe the circadian system and photoentrainment for *D. melanogaster*.

Besides CRY that is located in the circadian clock neurons themselves, fruit flies use the rhodopsins in their photoreceptive organs (compound eyes and ocelli) and their extra retinal eyelets, the Hofbauer–Buchner (HB) eyelets, for photoentrainment (reviewed in Senthilan et al. 2019). As light is able to penetrate the fly cuticle, the clock can directly be entrained by the HB eyelets and CRY even in the absence of all eye structures (Rieger et al. 2003). Only after elimination of CRY and all eye structures, entrainment to light–dark cycles is abolished (Helfrich-Förster et al. 2001). Nevertheless, such “circadian blind” flies still respond to light, indicating that additional photopigment(s) influence their activity. The search for these led to the detection of a seventh rhodopsin, Rh7, that mediates light responses, although there are diverging results and hypothesis concerning this finding (Senthilan and Helfrich-Förster 2016; Kistenpfennig et al. 2017; Ni et al. 2017; Baik et al. 2017; Grebler et al. 2017). Since the putative role of Rh7 in photoreception was recently reviewed in detail (Senthilan et al. 2019), I will largely skip Rh7 here, but instead focus on the light input pathways from the compound eyes and the HB eyelets to the circadian clock neurons and their putative interaction with CRY.

### Input pathways from the eyes and the HB eyelets to the clock neurons

Each fly compound eye consists of ~ 800 ommatidia, each of which contains eight receptor cells, six outer and two inner ones. The outer six receptor cells (R1–6) project into the lamina, where they connect to lamina monopolar neurons that run into the medulla, while the inner receptor cells (R7 and R8) project directly into the medulla (Fig. 2; Behnia and Desplan 2015). In contrast to this complex organization, each HB eyelet consists of only four receptor cells, located at the posterior edge between lamina and compound eye, that all project along the anterior surface of the medulla directly into the AME (Fig. 2). The somata of the clock neurons are located in the lateral and dorsal brain and their neurites are extensively connected with each other (Helfrich-Förster 2017; Top and Young 2018). Most of the clock neurons send dendrites into the AME, where they get direct light input from the HB eyelets and indirect light input via interneurons from the compound eyes (Fig. 2; Schlichting et al. 2016; Li et al. 2018). All photoreceptor cells use histamine as neurotransmitter, but the HB eyelets contain additionally acetylcholine and there is first evidence that they signal via acetylcholine to the s-LN_v_ and via histamine to the l-LN_v_ (Fig. 2; Schlichting et al. 2016). By patch-clamp recordings of the clock neurons, Li et al. (2018) could show that light from the eyes excites the great majority of clock neurons and that laser ablation of the AME abolishes the responses of the clock neurons to light. This clearly indicates that the AME serves as a kind of hub for light input from the eyes to most clock neurons, although this does not mean that all the clock neurons that get this light input contribute equally to behavioural entrainment.

### Organization of the circadian clock network with a special reference to the PDF neurons

As in cockroaches, crickets, beetles and bees, PDF neurons play a prominent role in the fly circadian system. In each brain hemisphere, four PDF neurons with small somata (called small ventrolateral neurons, s-LN_v_) and four PDF neurons with large somata (called large ventrolateral neurons, l-LN_v_) can be distinguished (Fig. 2). These two sets of PDF neurons have different projections and functions in the circadian clock of the fly (reviewed in Helfrich-Förster 2014, 2017; Top and Young 2018). The s-LN_v_ have dendrites in the AME and project into the dorsal brain. They communicate with the other clock neurons, especially with those located in the dorsal brain (dorsal clock neurons, DN), but also with more dorsally located lateral neurons (dorsolateral neurons, LN_d_). In addition, the s-LN_v_ appear to signal to neurons downstream of the clock (see Nagy et al. 2019 for a most recent report). Regarding locomotor activity rhythms, the s-LN_v_ are essential for circadian rhythms under constant darkness and, under light–dark cycles, they control the morning activity of the flies (fruit flies exhibit activity in the morning and evening with a siesta during midday; reviewed by Yoshii et al. (2012)). Therefore, they are also called morning cells (M cells, Fig. 2). Nonetheless, the s-LN_v_ do not work in isolation but cooperate with the DN and LN_d_ (see Fujiwara et al. 2018 and Chatterjee et al. 2018 for recent examples). The l-LN_v_ have also excessive dendrites in the ipsilateral AME that extend ventrally into the so-called ventral elongation of the AME. All four l-LN_v_ project via the posterior optic commissure to the contralateral optic lobe (Fig. 2). Three of them have net-like varicose arborizations in the entire distal left and right medulla, while one l-LN_v_ restricts its arborizations to the proximal part of both medullae (Schubert et al. 2018). Although the varicose network of PDF fibres is close to the terminals of the inner photoreceptor cells (e.g. R8 in Fig. 2), there appears no direct innervation from these receptor cells to the l-LN_v_ (Alejevski et al. 2019). Nevertheless, l-LN_v_ get light input from the L2 lamina monopolar cells that are downstream of the outer photoreceptor cells (R1-6) in the retina in addition to getting direct light input from the HB eyelets (Muraro and Ceriani 2015; Schlichting et al. 2016). In addition, they and several other clock neurons get light input from two peptidergic interneurons that are located between lamina and medulla, arborize in the lamina and send their axons directly into the AME (Li et al. 2018; Fig. 2). Among the clock neurons, the l-LN_v_ are thought to be especially devoted to transfer light information to the circadian system, because they mediate light-dependent arousal and wakefulness of the flies (Sheeba et al. 2008a, b; Parisky et al. 2008; Shang et al. 2008). Not only the eyes, but also CRY and perhaps even Rh7 that seem both present in the l-LN_v_ confer light sensitivity to these neurons (Fogle et al. 2011, 2015; Ni et al. 2017; Baik et al. 2017, 2019). CRY and Rh7 influence the excitability of the membrane and enhance action potential firing of the l-LN_v_ in response to blue and UV light. In spite of the high responsiveness of the l-LN_v_ to light, flies with silenced l-LN_v_ and mutated CRY can still entrain to light–dark cycles, clearly indicating that light input from the eyes to the clock neurons works via parallel pathways (Li et al. 2018). The l-LN_v_ and PDF may rather have a coordinating function in photoentrainment. Indeed, PDF from the l-LN_v_ (and s-LN_v_) strongly affects the other clock neurons (Seluzicki et al. 2014; Yoshii et al. 2009; Guo et al. 2014, 2016). It accelerates the molecular clock in the s-LN_v_ and slows it down in the LN_d_ and other clock neurons. Furthermore, PDF delays Ca^2+^ rhythms in the majority of clock neurons (Liang et al. 2016, 2017). In this respect, the action of PDF on the LN_d_ is especially interesting because the LN_d_ control the evening activity of the flies and are, therefore, also called evening neurons (E cells, Fig. 2). PDF is necessary to delay the Ca^2+^ rhythms in the LN_d_ from the morning to the afternoon so that the LN_d_ can control the activity increase in the evening (Liang et al. 2017). Under long summer days, the siesta extends and evening activity occurs later than under short days or at equinox, and this delay is caused by secretion of PDF from the l-LN_v_ (Menegazzi et al. 2017; Schlichting et al. 2019b; see also below). Although the l-LN_v_ have no projection toward the dorsal brain, they appear to affect the other clock neurons via PDF secretion into the AME (Choi et al. 2012; Helfrich-Förster 2014).

### Role of the different rhodopsins in circadian entrainment

The six outer receptor cells of the compound eyes express rhodopsin 1 (Rh1), which has a broad sensitivity to blue-green light, whereas the inner receptor cells can be divided into two subtypes. Either receptor cell 7 (R7) expresses the ultraviolet (UV)-sensitive rhodopsin 3 (Rh3) and receptor cell 8 (R8) the blue-sensitive rhodopsin 5 (Rh5), or R7 contains rhodopsin 4 (Rh4) that is sensitive to longer UV wavelengths and R8 the green-sensitive rhodopsin 6 (Rh6) (see Sancer et al. this issue). The four HB-eyelet cells express Rh6 and the ocelli express rhodopsin 2 (Rh2), which is present neither in the compound eyes nor in the HB eyelets.

Saint-Charles et al. (2016) tested the re-entrainment of different rhodopsin mutants to 8 h phase-advances and -delays of low-intensity light–dark cycles and found that four of the six rhodopsins can mediate re-entrainment: Rh1, Rh3, Rh4 and Rh6. No re-entrainment was found when all rhodopsins except Rh2 were eliminated, suggesting that the ocelli alone are not able to entrain the clock, at least not to dim light. Similarly, the Rh5-positive R8 cells alone were not able to entrain the flies to dim light. Most interestingly, Rh5, Rh6 and Rh1 can employ alternative phototransduction that is independent of the common phospholipase C and that works at medium and high light intensity (Szular et al. 2012; Ogueta et al. 2018). Thus, Rh5 might work exclusively via this alternative phototransduction. This pathway appears important because it finally targets the clock neurons that control morning and evening activity of the flies. Alejevski et al. (2019) demonstrated a prominent role of the Rh6-positive R8 cells in entrainment: all inputs from outer and inner receptor cells appear to converge to these Rh6 cells to contribute to circadian entrainment. This finding fits to the results of Schlichting et al. (2014), who found a prominent role of the Rh6-expressing inner receptor cells 8, in addition to the Rh1-expressing outer photoreceptor cells, in moonlight detection. It is also consistent with Ogueta et al. (2018), who found that the inner receptor cells 8 synchronize the s-LN_v_ to light dark cycles even in absence of CRY and without a functional phototransduction cascade in the other photoreceptor cells. While the anatomical connection between Rh6-positive receptor cells and the clock neurons is still unknown, there appear to exist two pathways connecting the outer photoreceptor cells with the clock neurons (see above). (1) The pathway via the L2 lamina monopolar cells to the l-LN_v_ (Muraro and Ceriani 2015) and (2) the pathway via the two peptidergic interneurons that arborize in the lamina and project into the AME (Li et al. 2018).

In summary, all photoreceptors and most rhodopsins of the compound eyes and the HB eyelets appear to contribute to entrainment, while the Rh6-positive receptor cells might play a prominent role in this process. It will be most interesting to reveal the precise input pathway from these photoreceptor cells to the clock neurons.

### Integration of the light inputs from rhodopsins and CRY in the clock neurons

So far, we have seen that multiple photoreceptors contribute to photoentrainment of *D. melanogaster* and the question arises how the clock neurons integrate these light inputs. Recent studies suggest that the diverse light-input pathways possess different light sensitivity and, therefore, may contribute with different weight to entrainment, just depending on the environmental conditions (Schlichting et al. 2019a). CRY is extremely sensitive and mediates entrainment at very dim light conditions obviously by temporal integration of photons (Vinayak et al. 2012). This is reminiscent of the large and extraordinary prolonged electrophysiological responses of mammalian melanopsin that integrates photons over a time course of at least minutes (Do et al. 2009). Nevertheless, the intrinsically photosensitive retinal ganglion cells require 10^4^–10^6^ fold more photons than cones or rods, respectively, to obtain half-saturating responses, most probably due to the low pigment density of melanopsin resulting in a low probability of photon capture. Consequently, melanopsin may mainly work at high light intensities, which is different from CRY that mediates entrainment at very low light intensity and is furthermore responsible for phase shifting the fly’s activity after the administration of short light pulses (Emery et al. 1998; Stanewsky et al. 1998; Kistenpfennig et al. 2012). In *Drosophila*, the HB eyelets are the extraretinal photoreceptors that contribute predominantly to entrainment at high-intensity light (Schlichting et al. 2019a), while the compound eyes are mainly mediating circadian entrainment under low- to middle-intensity light conditions (Ogueta et al. 2018).

Light of different intensity does not only recruit different photoreceptors for clock photoentrainment; light does also change the dominance of the clock neurons in controlling rhythmic activity (Chatterjee et al. 2018; Schlichting et al. 2019b). As mentioned above, flies are mainly active in the morning and evening and exhibit a siesta during midday. Chatterjee et al. (2018) found that the morning activity-controlling s-LN_v_ cooperate with a group of dorsal neurons in the absence of light and swap their partner oscillator to the evening activity-controlling LN_d_ in the presence of light. When exposure to light further increases, the light-activated LN_d_ neurons finally become independent from the s-LN_v_ (but dependent on the l-LN_v_ as exemplified below). A main driver for these switches in coupling and clock neuron dominance is a light-driven increase in PDF expression and secretion from the s-LN_v_. Schlichting et al. (2019b) found a further light-mediated circuit switching depending on PDF when flies adapt their activity to long summer days. With increasing photoperiods, flies extend their siesta by advancing morning activity and delaying evening activity (in other words, keeping morning activity close to dawn and evening activity close to dusk). PDF is necessary to provoke this behaviour (Yoshii et al. 2009; Liang et al. 2016; Menegazzi et al. 2017), but obviously this PDF does not come from the same neurons under short and long days. Under 12-h photoperiods, HB eyelet and R8 photoreceptor cells signal specifically to the s-LN_v_ and the s-LN_v_ signal then via PDF to the LN_d_ (see above). Under long photoperiods, a light-mediated circuit switch happens making the R8 photoreceptor cells signal predominantly to the l-LN_v_ and the latter to overtake the signalling to the LN_d_ (Schlichting et al. 2019b). The situation is even more complicated because CRY appears to buffer the eye-mediated phase-delaying effect of light on evening activity under long days (Kistenpfennig et al. 2018). Flies without CRY delay their evening activity even more than wildtype flies, showing that CRY keeps evening activity in the day and prevents it to shift toward or even into the night. Vice versa, flies without eyes or without PDF have an early evening activity, because they lack the phase-delaying effect of the eyes and PDF and, therefore, the phase advancing effect of CRY dominates. Thus, the interaction between the eyes and CRY balances the phase of their evening activity. At the same time, the complex and plastic control of evening activity timing by different photoreceptors and clock neurons enables the flies adapting in a flexible manner to diverse light conditions.

## Conclusions

Synchronization of circadian clocks with the external environment (also called circadian entrainment) is essential for their adaptive function and provides a critical link between the environment and the clocks. Circadian entrainment is a complex task, which is reflected in the number of involved photoreceptor pigments and organs. Most if not all animals involve their eyes, but in addition they use specialized photoreceptors that convey information about environmental light conditions to the circadian system. A very recent paper suggests that the situation is even more complicated (Lazopulo et al. 2019). This paper shows that photoreceptors in the eyes and the body wall of *Drosophila melanogaster* cooperate in mediating time-dependent colour preference and light avoidance. As we have seen in the fruit fly, the different photoreceptors may have slightly different tasks and may contribute differently to entrainment, just depending on the light conditions (low- or high-intensity light, short or long photoperiods). This enables the circadian clock of these insects to respond in a very plastic manner to the environmental light conditions. The fruit fly may be no exception in this flexibility and it will worth studying other insects in this respect.

## Abbreviations

ALA: Accessory laminae
AME: Accessory medullae
CRY: Cryptochrome
HB eyelet: Hofbauer–Buchner eyelet
LN_d_: Dorsal lateral neurons
LN_v_: Ventral lateral neurons
s-LN_v_: Small ventral lateral neurons
l-LN_v_: Large ventral lateral neurons
PER: Period protein
PDF: Pigment-Dispersing Factor
R7: Receptor cell 7 of the compound eye
R8: Receptor cell 8 of the compound eye
Rh: Rhodopsin
SCN: Suprachiasmatic nucleus
TIM: Timeless protein
